# Effectiveness of immunization with multi-component bacterial immunomodulator in foals at 35th day of life

**DOI:** 10.1038/s41598-022-17532-1

**Published:** 2022-09-22

**Authors:** Migdał Anna, Migdał Łukasz, Okólski Adam, Anna Chełmońska-Soyta

**Affiliations:** 1 Department of Genetics, Animal Breeding and Ethology, Faculty of Animal Sciences, University of Agriculture in Krakow, al. Mickiewicza 24/28, 30-059 Kraków, Poland; 2 University Centre of Veterinary Medicine UJ-UR, University of Agriculture in Krakow, al. Mickiewicza 24/28, 30-059 Kraków, Poland; 3 Laboratory of Reproductive Immunology, Hirszfeld Institute of Immunology and Experimental Therapy, Polish Academy of Sciences, Weigla 12 Street, 53-114 Wroclaw, Poland; 4 Department of Immunology, Pathophysiology and Veterinary Preventive Medicine, Division of Immunologyand Veterinary Preventive Medicine, Faculty of Veterinary Medicine, Wroclaw University of Environmentaland Life Sciences, Norwida 31 Street, 50-375 Wroclaw, Poland

**Keywords:** Zoology, Animal physiology

## Abstract

The aim of the study was to investigate the mechanisms leading to immunization through the use of a multicomponent bacterial immunomodulator and to find out the relationship between the TLR 4 receptor with selected parameters of innate immunity and to acquire immunity. The study was conducted on 18 Polish Pony Horses foals divided into two study groups: control (n = 9) and experimental (n = 9). Foals from the experimental group received intramuscular duplicate injection of 5 ml of multi-component bacterial immunomodular at 35 and 40 days of age. RNA isolated from venous blood was used to evaluate the expression of *TLR4* genes using RT-PCR. Concentration of Il-6, IL-10, IgM and IgG2 was determined by the ELISA method in blood plasma. Immunostimulation had a significant impact on the level of genes expression for *TLR4* expression and IL-6 concentration. No effect of stimulation on IgM and IgG2 concentrations was found. The expression of *TLR4* genes as well as the levels of interleukins could be modulated by stimulation with a pharmacological agent multi-component bacterial immunomodulator. The experiment demonstrated a strong positive correlation between TLR4 gene expression and IL-6 concentration and between TLR4 gene expression and IgM concentration.

## Introduction

A foal is born capable of an immune response, although at the time of birth it is almost devoid of immunoglobulins. Immediately after birth, only insignificant amounts of IgM and IgG1 can be detected in the blood of newborns^1^. Immediately after birth, foals have 30% of the number of B cells found in adult animals. The efficiency of T lymphocytes and neutrophils as well as the activity of the complement system are also lower^2^. In the first days of life, along with increasing exposure to a variety of pathogens, foals respond with a massive expansion of antigen-specific lymphocytes. This response is the result of a progressively increasing number of circulating lymphocytes and the similarly growing mass of peripheral lymphoid organs^3^. Endogenous antibody production at 5–8 weeks of age is minimally sufficient to protect the foal (> 40–50 mg/dl). Meanwhile, the concentration of colostral IgG decreases with the half-life thereof, which is 28–32 days in healthy foals. In foals deprived of colostrum and fed with replacement preparations, the level of endogenous immunoglobulins increases significantly at 2 weeks of age, and at 5 weeks it is comparable or higher than the level of immunoglobulins in the blood plasma of foals fed with mare’s milk. In cow colostrum-fed foals, the production of endogenous antibodies is not affected^4^. Neonatal and adult equine B lymphocytes show differences in the profiles of the immunoglobulin G subclasses and immunoglobulin A production. B lymphocytesin newly born foals mainly produce IgG1 and IgG3, while endogenous IgG2 and IgG4 are absent. Adults, on the other hand, produce more IgG2 and IgG4^5^. The concentration of IgG_4/7_ (IgGb) in foal blood plasma is variable, and its production appears to be delayed in response to toxins, viruses, and intracellular bacterial infections^6–8^. Although the production of IgG1 (IgGa), IgG_3/5_ (IgGT) and IgA already in the first 8–12 weeks of foal life is similar to that of adult horses, the concentration of IgG_4/7_in foals up to 1 year of age remains lower than in adult horses^9^. The deficiency of some subclasses of immunoglobulins can lead to the impairment of the body’s functional protection. Certain genes responsible for the production of immunoglobulins, in particular with IgG4 and IgG7, may have different expression and regulation in foetuses and young foals compared to adult horses^5^. Despite the fact that foals synthesize immunoglobulins in utero, their concentration does not reach the minimum level necessary to provide protection against pathogens by the age of 2 months. In the first weeks of life, the production of endogenous immunoglobulins and the immune response of foals are inhibited by the still present, passively transmitted maternal antibodies. It is difficult to precisely define the period in which the maternal immunity is active, as it depends on a number of factors. The disappearance of passive maternal immunity in a newborn foal occurs about 2–6 weeks after birth^10^. The emerging and increasing resistance to antibiotic therapies forces the search for newer and more effective preventive methods and new animal treatment strategies. It seems that an alternative is the prophylactic immunostimulation of foals before weaning from the mother. There is a growing interest in this type of preparations, which are increasingly often applied in veterinary practice, with promising results. Among immunostimulants, there are compounds of natural and synthetic origin. Natural immunostimulants include compounds of bacterial origin, substances isolated from fungi and from plants. Bacterial stimulants include whole bacterial cells, live bacteria of the genera *Bifidobacterium* and *Lactobacillus*, belonging to the group of organisms called probiotics. There are also bacterial extracts and bacterial products with an immune-stimulating effect, including OK 432 (Picibanil), obtained from *Streptococcus pyogenes* and *Streptococcus heamolyticus* strains with low virulence, lipopolysaccharides (LPS), which are part of the cell wall of Gram-negative bacteria, as well asmuramyl dipeptide (MDP) and its analogues^11–15^. Often, immunotherapy is associated with side effects including injection site reactions, fever, somnolence, and decreased appetite. This is probably related to the induced endogenous release of cytokines. It is desirable to stimulate the immune response without harmful inflammation and tissue damage. The choice of immunomodulators and recommendations for action are still questioned in many cases due to insufficient information on the mechanisms of action in vitro and in vivo, as well as unknown harmful side effects^16^. The innate immune response is a universal defence mechanism against invading pathogens. Toll-like receptors belonging to this line of defence are involved in the recognition of a wide range of microorganisms: bacteria, viruses, and fungi. Nevertheless, few authors dealt with the issue of gene expression for Toll-like receptors in the neonatal period of foals, and there is little information on the shaping of expression of these genes in other farm animals.

So far little is known about the development of the horse immune system during pre- and postnatal periods, which negatively affects devising strategies for maintaining and improving foal health. Based on the knowledge available in the literature, biological properties and the influence of Toll-like receptors on the characteristics of the immune response of farm animals and humans, we hypothesized that the expression of TLR-4 receptor genes and the level of cytokines and immunoglobulins in foals depend on a factor such as bacterial immunostimulation, and especially LPS immunostimulation.

The aim of the study was to investigate the mechanisms leading to immunization through the use of a multicomponent bacterial immunomodulator and to find out the relationship between the TLR 4 receptor with selected parameters of innate immunity and to acquire immunity after stimulating the immunity with a bacterial preparation in foal up to 60 days of age.

## Results

### Influence of stimulation on expression of TLR4 mRNA

**Figure 1:**
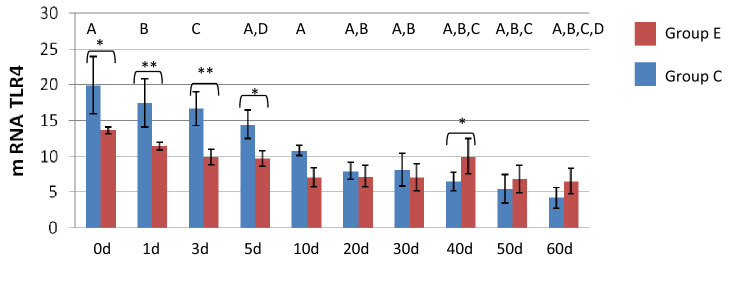
Trends in changes of TLR4 gene expression after immunization. 0d—sample collected at delivery; 1d—sample collected 24h after delivery; 3d—sample collected 3rd days after delivery; 5d—sample collected 5th days after delivery; 10g—sample collected 10th days after delivery; 20d—sample collected 20th days after delivery; 30d—sample collected 30th days after delivery; 40d—sample collected 40th days after delivery; 50d—sample collected 50th days after delivery; 60d—sample collected 60th days after delivery. Means are reported with their standard errors Group C—control group; Group E—experimental group stimulated multi-component bacterial immunomodulator (injection in 35th and 40th days after delivery). *Means in row/line for receptor show significant differences (*p*<0,05). **Means in row/line for receptor show highly significant statistical differences (*p*<0,01). Means for control group with same letter show highly statistical significant difference (*p* < 0.01).

Before the injection of the immunomodulator, both groups showed a similar trend in the level of *TLR4* mRNA expression. In control groups, a dynamic decrease in the level of expression was observed (Fig. 1). The highest level was observed in samples taken before the first suction. In the first 30 days of foal life, a higher level of expression was noted in group C. These differences were highly statistically significant (Fig. 1). 5 days after the first dose multi-component bacterial immunomodular injection *TLR4* mRNA expression increased by 41.33% (group E), while expression of *TLR4* mRNA in foals from group C decreased by 20.22%. 15 days after the injection, the expression of the studied gene in the experimental group decreased, but was still at a higher level than in the control group. On the following days after immunostimulation, *TLR4* mRNA expression in foals from group E was higher but no statistical differences were found between groups. Statistical analysis showed statistically significant differences in *TLR4* mRNA expression after immunostimulation, in group E between 30 and 40th days of life and at 40th days of life between groups.

Sperman's rang correlation test showed strong positive correlation between *TLR4* gene expression and level of lymphocytes.

### Influence of stimulation on immunoglobulins concentration

**Figure 2:**
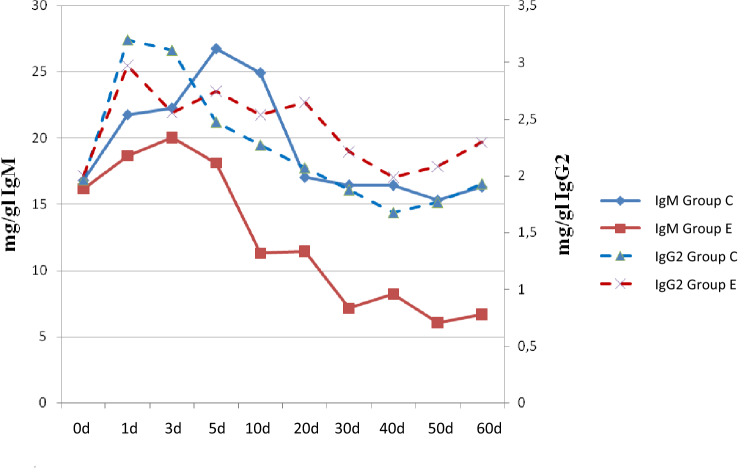
Influence of immunization on IgM and IgG2 concentration. 0d—sample collected at delivery; 1d—sample collected 24h after delivery; 3d—sample collected 3rd days of life; 5d—sample collected 5th days of life; 10g—sample collected 10th days of life; 20d—sample collected 20th days of life; 30d—sample collected 30th days of life; 40d—sample collected 40th days of life; 50d—sample collected 50th days of life; 60d—sample collected 60th days of life Group C—control group; Group E—experimental group stimulated multi-component bacterial immunomodulator (injection in 35th and 40th days after delivery). IgG2 means at 30th, 40th, 50th and 60th days of life show significant differences with Kruskal-Wallis test (*p*<0,05). IgG2 means at 3rd and 20th days of life show highly significant statistical differences Kruskal-Wallis test (*p*<0,01). IgM means at 5th days of life show significant differences with Kruskal-Wallis test (*p*<0,05). IgM means at 30th, 40th, 50th and 60th days of life show highly significant statistical differences Kruskal-Wallis test (*p*<0,01).

The changes in the concentration of IgG2 observed during the course of the experiment followed the same pattern in both groups. The highest IgG2 concentration in foals was reported 24 h after the delivery (group C; 3.20 ± 1.21 mg/dl). In the both control and experimental groups, the lowest concentrations were also observed at day 40 (1.68 ± 0.32 mg/dl and 1.99 ± 0.28 mg/dl, respectively). On the remaining days, concentration of IgG2 decreased in both groups (Fig. 2). Statistical analysis revealed highly significant differences (*p* < 0.01) in IgG2 concentration between groups C, E at 24 h, 3, 5 10, 20 and 30 days of age foals. As well statistically significant differences (*p* < 0.05) between study groups were shown at 40, 50 and 60 days of age. Spearman’s rank correlation analysis showed a strong positive correlation between the mean daily IgG2 concentration and the mRNA expression of genes for *TLR4* (R = 0.58).

The dynamics of changes in the concentration of immunoglobulin M in both groups was similar (Fig. 2). In the first days of foal life, the IgM concentration increased, reaching the highest values between the 3rd and 5th day after birth. The highest level was observed on the 5th day of life in foals from the C group: 26.75 ± 3.76 mg/dl. In the following days, a decrease in the concentration of these antibodies was observed, with the lowest concentration on the 50th day of life in foals from the E group: 6.04 ± 1.85 mg/dl. At 60 days of foal life in all groups, a slight increase in immunoglobulin M concentration was observed. Statistical analysis showed highly significant differences (*p* < 0.01) between groups C and E between the 30 and 60th day of foal life. There were also significant differences (*p* < 0.05) in the results obtained on day 5, between group C and group E. Spearman’s rank correlation analysis showed a strong positive correlation between the mean daily IgM concentration in all groups, and the mRNA expression of the genes for the *TLR4* receptor (R = 0.52).

### Influence of stimulation on cytokins concentration

**Figure 3:**
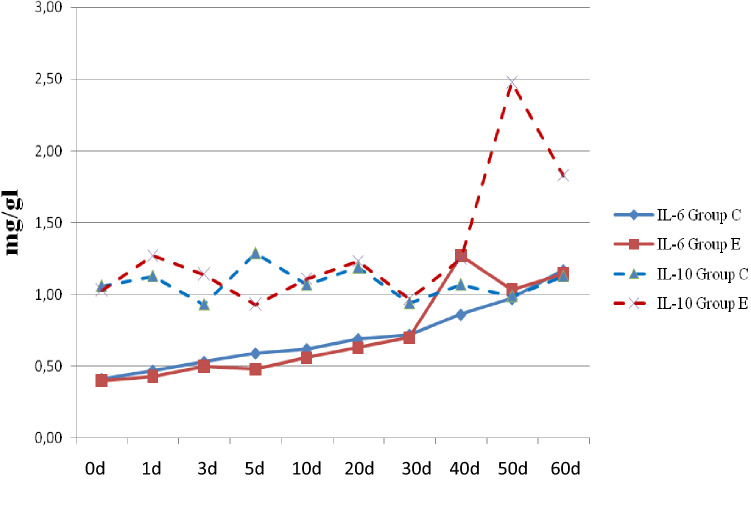
Influence of immunization on IL-6 and IL-10 concentration. 0d—sample collected at delivery; 1d—sample collected 24h after delivery; 3d—sample collected 3rd days of life; 5d—sample collected 5th days of life; 10d—sample collected 10th days of life; 20d—sample collected 20th days of life; 30d—sample collected 30th days of life; 40d—sample collected 40th days of life; 50d—sample collected 50th days of life; 60d—sample collected 60th days of life. Group C—control group; Group E—experimental group stimulated multi-component bacterial immunomodulator (injection in 35th and 40th days after delivery). IL-6 means on the 40th days of life show highly significant statistical differences with Kruskal-Wallis test (*p*<0,01). IL-10 means on the 50th and 60th days of life show highly significant statistical differences with Kruskal-Wallis test (*p*<0,01).

The profile of changes in interleukin 6 concentration in all groups was similar (Fig. 3). In the first days of foal life, the concentration of IL-6 has shown a very low level, and during the birth it was at a level that was borderline mark able (0.4 ± 0.03 mg/dl in foal group C. In the following days there was a steady increase in IL-6 concentration in both groups, whereas in the control group. In the experimental group of the foals, of IL-6 was recorded on the 40th day of foal life 1.27 ± 0.11 mg/dl and it was the highest observed concentration. The analysis showed a highly statistically significant effect of immunostimulation on the concentration of IL-6.

The conducted experiment did not show any statistically significant changes in the concentration of interleukin-10 between the studied groups. The lowest concentration of IL-10 was observed during delivery, 0.93 ± 0.12 mg/dl, and the highest, on the 60th day of foal life, 1.13 ± 0.14 mg/dl, respectively. In the group of foals subjected to immunostimulation (group E), a higher concentration of IL-10 was observed. In this group, the lowest level was observed on the 5th day of life: 0.93 ± 0.10 mg/dl; and the highest on the 50th day: 2.48 ± 0.35 mg/dl. Statistical analysis showed highly significant differences between the study groups in IL-10 concentration (*p* < 0.01) on the 50 and 60th day of foal life. Spearman’s rank correlation analysis showed a weak positive correlation between the mean daily IL-10 concentration and the mRNA expression of genes for *TLR4* (R = 0.14).

## Discussion

Despite the availability of newer and more accurate diagnostic, preventive and therapeutic methods, viral and bacterial infections in foals still constitute a major problem in horse breeding. Sepsis remainsa major cause of deaths in newborn foals^17^. In our own research, a highly significant effect of immunostimulation on the level of gene expression for the Toll-like 4 receptor was observed. Vendrig et al.^18^ also failed to show the effect of LPS stimulation on the expression level of the studied gene in foals, in contrast to adult horses, in which increased expression was observed under the influence of stimulation. In this study used PBMCs from adult horses and newborn foals (< 12 h postpartum)^18^. Different results were obtained by Tessier et al.^19^, who compared the *TLR4* gene expression of cord blood multipotent mesenchymal stromal cells and peripheral blood cells MSC. The authors reported a strong increase in the expression of the studied gene in response to stimulation with lipopolysaccharides (LPS). Hansen et al.^20^, having studied horses (isolated PBMCs) aged 5 to 27 years, showed an increase in the level of expression after LPS stimulation, while no reaction after stimulation with PGN (peptidoglycan derived from *Staphylococcus aureus* extract) was reported. Lin et al.^21^, who examined the level of gene expression for *TLR4* in pigs lungs tissues, reported a higher value in animals with bacterial *Streptococcus suis* infection and H1N1 viral infection, but a significantly higher value of expression was observed in animals with mixed bacterial-viral infection. The studies of the effect of immunostimulation on the expression of the gene for the Toll-like 4 receptor in humans were investigated by Levy et al.^22^ and Yerkovich et al.^23^. These studies showed that the level of *TLR4* gene expression in the peripheral blood of LPS-immunostimulated neonates was higher compared to non-stimulated neonates and adults. In our own study in the first days of foals life, significant differences between the groups on the level of gene expression for the Toll-like 4 receptor was observed. Significant differences in lymphocyte levels were observed on the same days. In our own studies, the level of *TLR4* expression was also dependent on the level of lymphocytes. This requires further investigation and extension of research methods, it has been suggested that dysbiosis of the intestinal microbiota activates *TLR4*. Horses, like humans, are sensitive to LPS and develop severe endotoxemia, a primary cause of death in cases of equine colic. Since little is known about *TLR4* signaling in equine B lymphocytes, the current study analyzed concentrations of cytokines produced by potential *TLR4* signaling pathways.

Foal Peripheral Blood Mononuclear Cells (PBMC) actively produce cytokines, including TNF-a, IL-1 beta, IL-2, IL-4, IL-6, IL-8, IL-10, IL-12p35, IL15, and IL-18, already in the first month of life. It has been proven that a small number of PBMCs are enough to increase the expression of IFN-γ, IL-4 and IL-10, and these proteins are detected after stimulation, already from birth^24,25^. Breathnach et al.^26^ and Wagner et al.^24^ observed an increase in IL-10 concentration during the first six weeks of foal life, followed by stabilization at a level similar to that of adult horses. In our own studies in stimulated foals, an increase in the level of IL-6 was observed along with an increase in the expression of the gene for *TLR4*, followed by an increase in IL-10. Although the results of analyses of cytokine levels in foals with sepsis are contradictory, this may reflect an immune response to various types of infectious organisms, or a transient immunosuppression. In the authors’ own research it was found that the level of IL-6 in the first days of foal life was very low, and it fact, it was borderline measurement possibilities. Similar results were obtained by Burton et al.^27^. However, the increase in IL-6 levels in the following days does not have to be related to endogenous production. In newborn foals, the situation seems to be more complicated. The massive transfer of IL-6 from colostrum into the bloodstream makes it difficult to assess endogenous production levels. Literature data indicate that mare’s colostrum, like the colostrum of other animal species, contains IL-6^28–31^. As a result, healthy foals that drank sufficient amounts of colostrum after birth have acquired a high concentration of colostral IL-6. This hypothesis was confirmed in calves^32^ and piglets^30^. While maternal IL-6 may influence the immune response in the newborn, its exact function in the foal’s immune system is still unknown. Our studies showed that the muli-components bacterial immunumomodulator activates the TLR4 receptor and the resulting upregulation of cytokine secretion. Our Spearman correlation analysis showed that significant types of differences were positively correlated with TLR4 and IL-10, suggesting activation of the TLR4/IL-10 signaling pathway of immunization. In our study, an increase in the concentration of immunoglobulin M was observed in the first 3–4 days of foal life, with subsequent decrease until the 50th day of life, when the lowest value was observed. No significant effect of immunostimulation on IgM levels was recorded. The increase in IgM concentration in the first days of life was also reported by Bondo and Jensen^33^. Flaminio et al.^34^ observed a higher concentration of IgM in blood samples taken shortly after delivery compared to blood samples taken in the second and third month of foal life. These concentrations, however, were lower than those observed in adult horses, which is consistent with the results of own research by Flaminio et al.^34^. In this experiment, no statistical differences were observed between 6 week-old *Rhodococcusequi*-infected versus healthy foals. These results are also confirmed by studies carried out on foals infected with *S. aureus*. IgM concentrations in the serum of healthy and infected foals were at an even level. The influence of age on the level of IgM was observed, as on the 30th day significantly lower concentrations were recorded compared to the results obtained on the first day of foal life^3^. Also studies by Ryan and Giguere^35^ showed significantly lower humoral immunity response and lower lymphoproliferation in response to vaccination in newborn foals compared to adult horses, even in the absence of vaccine antigen-specific antibodies. In these studies, the vaccine immune response increased with age, yet 3 month-old foals did not yet respond with the same intensity as adult horses^35^. In newborn foals, no increase in the level of immunoglobulins after immunostimulation was demonstrated, only in 3 month-old foals an increase in IgM and IgG_4/7_ was observed, which is confirmed by the results obtained in our own research. Jacks et al.^36^ reported different results, because after vaccinating 7 day-old foals with live *Rhodococcusequi*, they observed a significant increase in IgM concentration. These results indicate that a humoral immune response can be stimulated in newborn foals. However, the nature, the dose of the antigen, and the type of adjuvant, if any, can all have a profound effect on the intensity of the response and the class of immunoglobulin produced. The specific functions for IgG2 are still poorly understood^37^. Few authors describe IgG2 in horses. So far, there are no reports on the level of this immunoglobulin in foals in the first weeks of life. The limited amount of information regarding the IgG2 function in foals is exacerbated by the impossibility of comparing it to other species of mammals, which is due to a different number of immunoglobulin G subclasses. In our study, a very low level of G2 immunoglobulins was observed, on borderline measurement possibilities, and no effect of stimulation on IgG2 concentration was found. Similar results were obtained by Lewis et al.^38^, who analysed the effect of stimulation with protein A obtained from *S. aureus* or with protein G obtained from *Streptococusequi* on the concentration of individual immunoglobulin G subtypes, and found a very weak IgG2 response. The authors suggest that for maximum efficacy, vaccination should target immunoglobulin G responses of the IgG1, IgG3, IgG4, and IgG7 subtype. The paradox of neonatal vaccination is the need for immediate protection in early life and obtaining long-term memory despite the limitations of the infant’s immune system and the theory of maternal antibody interference.

In summary on the basis of the results obtained, it was concluded that the expression of genes of *TLR4* genes as well as the levels of interleukins could be modulated by stimulation with a pharmacological agent multi-component bacterial immunomodular. The experiment demonstrated a strong positive correlation between *TLR4* gene expression and IL-6 concentration and between *TLR4* gene expression and IgM concentration. Detailed knowledge of the molecular mechanisms of immunglobulin synthesis appears necessary for a better understanding of foal immunity maturity and its contributing factors. At the same time, it encourages studies regarding the influence of signalling cascade’s proteins on the primary immunological response, which provides an opportunity to develop extremely precise methods of regulating the acquired immunity. There is still little information about the maturity of the horse’s immune system in the pre- and postnatal period, which negatively affects the planning of foals’ health protection strategies.

## Methods

### Animals and feeding

Studies were carried out on 18 foals representing Polish Pony (Konik, Polish Konik) horses. This primitive horse breed is genetically and phenotypically closely related to its wild ancestor, the Tarpan Horse (Euroasian wild horse)^39^.

All foals with mares were kept in the same stable in individual boxes (size 2.15 × 3.50 m) on permanent straw bedding at the Experimental Station of the University of Agriculture in Krakow. All animals were clinically healthy throughout the experimental period. Mares of 5 to 17 years of age, and 270 to 340 kg live body weight, were not vaccinated during pregnancy. Foal birth weight was 27–35 kg, weight loss on the first day of life was < 1.5%.The horses had all been used by university students in the teaching program. No horses were used for equestrian purposes. Inclusion criteria consisted of foals born from healthy mares with no placentitis, normal gestational period, uneventful birth, and having normal physical and neurological examination findings. The foals had to successfully stand and nurse within 2 h of birth, and remain clinically healthy during the study period.

Mares were fed ad libitum with hay (*Lolium* 40%, *Trifolium* L.20%) with addition of oat in the amount of 1.5 kg/mare per day (according to Institute of Physiology and Animal Nutrition standards, 1997)^40^. Foals were fed only with colostrum and mother’s milk ad libitum, without additional supplementation. Water was offered from automatic water drinkers (flow ~ 10 l/min).

### Experiment design

320 days of pregnancy, birth alarm (*Abfohlsystem, Jan Wolters*) was placed in the labia, and mares were moved to box stalls inside a stable, lit with natural light (Supplementary Fig. S1). During the experiment, the foals were kept with their mothers in individual boxes, and were leaving the stalls with the mothers for pasture. The following physiological parameters determined the selection of animals for the experiments:

- Blood counts in foals on the first day of life (hematocrit > 40%, hemoglobin > 13 g/dL, the level of erythrocytes 9.5 × 106/µl, leukocyte level > 6 × 103/µl).
- The quality of the colostrum before the first suckling (immunoglobulin concentration > 30 g/dL, > 20% BRIX).
- Evaluation of the foal's viability in the first 5 min. after birth (obtaining min. 8 pts on the Apgar scale).

Foals were randomly assigned into two groups:

- Experimental Group (E; n = 9)—foals that received the multicomponent bacterial immunomodulator; and
- Control Group (C; n = 9)—foals that did not receive the multicomponent bacterial immunomodular or any other pharmacological/additive component that might influence their immune.

For immunostimutalion used in the present study, multi-component bacterial immunomodulator. It consisted of a mixture of inactivated gram-positive and gram-negative bacteria e.g.: *Escherichia coli* (123 mg/ml), *S. aureus* (74 mg/ml), *Streptococcus zooepidermicus* (24.6 mg/ml), *Streptococcus equi* (24.6 mg/ml), *Streptococcus equisimilis *(24.6 mg/ml), *Streptococcus agalactiae* (24.6 mg/ml), *Streptococcus dysgalactiae* (24.6 mg/ml), *Pasteurellamultocida* (123 mg/ml), and *Erysipelothrixinsidiosa* (49 mg/ml) as well as pork spleen extract (10 mg/ml). On day 35 and day 40 after birth, the foals from the experimental group received intramuscular (*m. pectoralisdescendens*) injection of 5 ml of multi-component bacterial immunomodulator.

### Blood sampling and analysis

Blood samples were collected from foals by jugular venipuncture. Blood samples were obtained from foals until 60 days of age according to the following scheme: before the first suckling, at 1st, 3rd, 5th, 10th, 20th, 30th, 40th, 50th and 60th day of age. Three mililitres of blood were collected into TEMPUS (Applied Biosystems, Tempus™ Blood RNA Tubes) tubes with RNA stabilizing factor. Samples were stored at − 20 °C till further processing. Three mililitres of blood were collected into tubes with EDTAk2. The collected samples were centrifuged at 5000 rpm for 5 min and plasma were stored in − 20 °C till further analysis.

### Isolation of RNA

Isolation of RNA was carried out using TEMPUS SPIN (Ambion) according to the manufacturer’s protocol (Supplementary). 1 µg RNA was transcribed into cDNA using High Capacity cDNA Reverse Transcription Kit (Applied Biosystems) according to the protocol.

A “No-RT” (non-reverse transcriptase) control was used for selected RNA samples to analyse DNA contamination in RNA samples.

### Gene expression analyses

Gene expression analyses were performed in theIllumina Eco (Illumina) system using TaqMan^®^MGB probes (Table 1). Every sample was analysed in triplicate in the final volume of 10 µl (Supplementary Table S1). Amplification was performed according to the following protocol: polymerase activation at 95 °C (2 min) and 40 cycles: 95 °C for 15 s and 60 °C for 1 min. SDHA and HPRT genes were used as housekeeping genes (Table 1).

**Table 1:** Probes used for amplification of TLR genes and housekeeping genes.

Gen	Full name of the gene	Access number GenBank	Taqman gene expression assay ID	Dye
*TLR4*	Toll-like receptor 4	NC_009168.2	Ec03468993_m1	FAM
*SDHA*	succinate dehydrogenase complex subunit A	XM_001490889	Ec03470479_m1	VIC
*HPRT*	Hypoxantine phosphoribosyl transferase	AY372182.1	Ec03470217_m1	VIC

In addition, an analysis of blood morphotic parameters was performed (Supplementary material).

### Immunoglobulins and cytokins level

Immunoglobulins and cytokins level were measured using ELISA assay in Spectramax Plus 384 Microplate Reader (Molecular Devices) using 96-well plates coated with monoclonal antibodies against equine IgG2 (GR106527, Genorise) IgM (GR106524, Genorise), IL-6 (GR106001, Genorise), and IL-10 (GR106003, Genorise) (Genorise Scientific, Devon-Berwyn, PA, USA) (Supplementary Table S3). 100 µl of plasma per well were added. Each well was washed three times with Assay Buffer after each step. 100 µl of working dilution of Detection Antibody were added and incubate 1 h. After wash 100 µl of working dilution of Conjugate were added to each well and incubated for 20 min. After the wash, 100 µl of Substarte Solution were added to each well, and incubated for 20 min. After the wash, 50 µl of Stop Solution were added to each well. Duplicate wells were used for each sample. Plates were read using microplate reader set to 450 nm. Results were calculated based on standard curve. High standard concentration of 20 ng /ml for IgG2 and 2520 ng /ml for IgM were prepared, vortexed for 15 s, and allowed to sit for 5 min. A seven-point standard curve was generated using twofold serial dilutions in the Assay Buffer.

### Statistical analysis

Data are presented as means ± standard error. The data were analysed using SAS 9.4 software (SAS Institute INC., USA). The Shapiro–Wilk test was considered the best test to check the normality of the distribution of a random variable. Because the data did not have normal distribution, Kruskal–Wallis test was used, with immunostimulant as effects and for interactions between group and time post-immunization on relative antibody concentration and cytokine and mRNA expressions. The relationships between all parameters (TLR4 mRNA expresion level, concetration of IL-10, IL-6 ang IgG2, IgM) were analyzed using nonparametric Spearman’s rank correlation test. The value ranges from 0.0 to 0.5, from 0.5 to 1.0, from − 0.5 to 0.0 and from − 1.0 to − 0.5 indicate weak positive, strong positive, weak negative and strong negative correlation, respectively.

### Ethics committee

The authors have declared: Experiment with animals were carried out according to the ARRIVE guidelines (McGrath et al. 2010); The protocol was approved by the Dean Faculty of Animal Sciences, University of Agriculture in Krakow (no 37, 30 May 2016); Veterinary care and samples collecting were performed by vet prof. Adam Okólski; All animals used in this study were help in experimental station of University of Agriculture in Krakow; All methods were carried out in accordance with relevant guidelines and regulations.

### Ethical approval

The experiment was conducted upon receiving the permission granted from the Local Ethics Committee in Krakow (No. 37, 30 May 2016).

## Supplementary Information

Supplementary Information.

## Data Availability

The datasets used and/or analysed during the current study available from the corresponding author on reasonable request.
