# Supplementary material for: Effectiveness of immunization with multi-component bacterial immunomodulator in foals at 35th day of life
Source: Sci Rep. 2022 Sep 22;12:15795. doi: 10.1038/s41598-022-17532-1 (PMC9499974; doi:10.1038/s41598-022-17532-1)
Supplement: Supplementary file 1 — Supplementary Information. [file 41598_2022_17532_MOESM1_ESM.doc]

# Effectiveness of immunization with multi-component bacterial immunomodulator in foals at 35th day of life

Migdał Anna^1*^, Migdał Łukasz^1^, Okólski Adam^2^, Anna Chełmońska-Soyta

**Supplementary material**

RNA was isolated from blood using TEMPUS SPIN (*Ambion*®) method. Isolated Total RNA was quantified on Nanodrop 2000 method (ThermoScientific, Wilmington, USA) and assessed qualitatively on 2% agarose gel with addition of ethidium bromide. 1 µg RNA was transcribed on cDNA in 37ºC using High Capacity cDNA ReverseTranscription Kit (*Applied Biosystems*) according to protocol. For some RNA samples “No-RT” was performed.

**Real-time PCR**

Gene expression analysis were performed on Illumina Eco (Illumina) using TaqMan®MGB probes with fluorescent labels FAM and VIC. For every sample reaction was performer in triple replication in 10µl volume. Mastermix were prepared according to protocol and contain: Master Mix GoTaq®Probe qPCR, cDNA, molecular probes 20xconcentarted TaqMan gene expression assay. Ultrapure water was added to receive the total volume of 10 µl (tab. S1). Reaction conditions were set according to GoTaq®Probe qPCR Master Mix protocol: polimerase activation (2 min in 95^o^C) and 40 cycles - 95^o^C by 15s (denaturation) and 60^o^C by 1 min (annealing/extension).

Tab. S1 Mastermix reaction

| Reagent | Volume (µl) |
| --- | --- |
| GoTaq®Probe qPCR Master | 5 |
| cDNA | 1.5 |
| TaqMan | 1 |
| H_2_O | 2.5 |

Using molecular probes with different fluorescent labels (FAM and VIC), multiplex reactions for every analysed gene were performed in presence of two housekeeping genes (SDHA and HPRT). Reaction efficiency for every genes was estimated using standard curve (tab. S2). Efficiency (E) was calculated according to equation E=10^[−1/slope]^ , from slope of obtained regression line. Relative number of transcripts was calculated according to formula: 1/E^(Ct)^, [E=efficiency (10^[−1/slope]^), Ct (threshold cycle)]

Tab. S2 RT- PCR reaction efficiency

| Gen | Full name  of the gene | Dye | Amplicon length (bp) | Reaction efficiency % |
| --- | --- | --- | --- | --- |
| *TLR4* | Toll-Like receptor 4 | FAM | 91 | 84 |
| *SDHA* | succinate dehydrogenase complex subunit A | VIC | 56 | 88,5 |
| *HPRT* | Hypoxantinephsophoribosyl transferase | VIC | 84 | 93 |


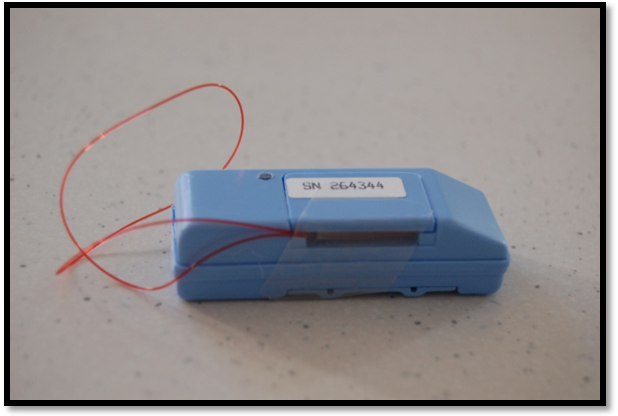

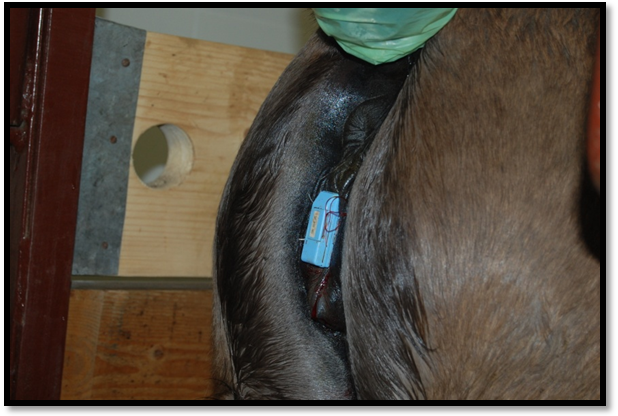


**A**

**B**

Fig. S1 Birth system alarm

1. Signal transmitter
2. Transmitter sewn in mare’s vulva

Tab. S3 Elisa reaction efficiency

| **Immunoglobulins and cytokines** | **Genorise Kit ID** | **Detection Range** | **Assay Sensitivity** |
| --- | --- | --- | --- |
| **IgM** | GR106524 | 0.39-25 ng/ml | 70 pg/ml |
| **IgG2** | GR106527 | 0.31-20 ng/ml | 60 pg/ml |
| **IL6** | GR106001 | 81-5200 pg/ml | 16 pg/ml |
| **IL10** | GR106003 | 310-20000 pg/ml | 25 pg/ml |

**Blood morphotic parameters analysis**

1 ml of blood was collected into EDTK3 tubes for hematology analysis. Tests were performed immediately after blood collections.

Erythrocyte counts were performed after 200-fold dilution of saline solutions using the Thoma chamber, leukocyte cells were counted after 20-fold dilution of Türk’s solutions at the Bürker chamber. The hematocrit was analyzed by the microhematocrit method by capillary centrifugation (2mins, 8000 rpm).

To perform a white blood morphology we prepared two blood smears, which were stained according to May Grünwald-Giemza methods (Mariańska 2006), and were analyzed using a stereoscopic microscope (SMZ 1000, Nikon, Japan over 1000 x). 200 leukocytes were found in the smear and were later divided into populations of lymphocytes, monocytes, neutrophils , basophils, intensive acid granulocytes (eosinophils).

Total hemoglobin was estimated by Drabkin’s cyanmethemoglobin method with a spectrometer SmartSpec BioRad.

Tab. S4 Level of blood morphotic elements over the subsequent days of age folas from control and experimental group (mean ± SE).

| Age  Parameters | | **<1h** | **24h** | **3d** | **5d** | **10d** | **20d** | **30d** | **40d** | **50d** | **60d** |
| --- | --- | --- | --- | --- | --- | --- | --- | --- | --- | --- | --- |
| Hematocrit (PCV) % | C | 50.00 ± 1.2 | 43.33 ± 1.2 | 39.83 ± 1.5 | 41.11 ± 1.1 | 41.56 ± 1.9 | 34.44 ± 1.6 | 37.33 ± 1.1 | 36.33 ± 1.3 | 37.72 ± 1.2 | 37.56 ± 1.7 |
|  | E | 50.50 ± 1.2 | 44.50 ± 1.8 | 43.67 ± 1.7 | 39.58 ± 1.5 | 41.17 ± 0.9 | 38.20 ± 1.1 | 39.75 ± 2.9 | 36.50 ± 1.7 | 37.25 ± 1.3 | 37.75 ± 0.9 |
| Hemoglobin (g/dl) | C | 15.46 ± 0.7 | 14.55 ± 0.7 | 13.26 ± 0.5 | 13.75 ± 0.7 | 13.63 ± 0.7 | 14.57* ± 1.2 | 15.11* ± 0.9 | 14.85* ± 1.3 | 13.30 ± 0.6 | 13.98* ± 1.1 |
|  | E | 13.94 ± 0.9 | 13.04 ± 1.7 | 13.50 ± 0.8 | 12.71 ± 0.6 | 12.39 ± 0.2 | 13.03 ± 0.9 | 11.80* ± 0.3 | 11.70* ± 0.4 | 12.39 ± 0.5 | 10.08* ± 0.3 |
| RBC count (10^6^/ µl) | C | 10.93 ± 0.9 | 10.62 ± 0.7 | 9.99 ± 0.6 | 10.55 ± 0.6 | 9.53 ± 0.4 | 10.38 ± 1.3 | 11.24 ± 1.5 | 9.98 ± 0.8 | 10.43 ± 0.8 | 9.86 ± 0.8 |
|  | E | 11.50 ± 0.5 | 10.01 ± 0.3 | 9.17 ± 0.4 | 10.24 ± 0.9 | 9.33 ± 0.8 | 9.04 ± 0.7 | 9.59 ± 1.0 | 9.69 ± 0.7 | 10.29 ± 0.3 | 10.22 ± 0.3 |
| WBC count (10^3^/µl) | C | 7.35 ± 0.8 | 7.71 ± 0.7 | 10.35 ± 1.2 | 9.86 ± 1.0 | 11.03 ± 0.8 | 11.30 ± 0.8 | 12.60 ± 1.0 | 14.31** ± 0.8 | 14.62* ± 0.7 | 13.07** ± 0.7 |
|  | E | 6.25 ± 0.6 | 8.17 ± 0.5 | 9.15 ± 0.8 | 10.64 ± 1.4 | 12.40 ± 1.1 | 12.48 ± 0.9 | 10.91* ± 1.6 | 21.75** ± 0.5 | 18.98* ± 0.4 | 14.89** ± 0.2 |
| Eozynophils (/µl) | C | 105** ± 2.2 | 116** ± 2.4 | 72** ± 1.5 | 296** ± 6.2 | 110** ± 2.3 | 226** ± 4.7 | 315** ± 6.6 | 286** ± 6.0 | 292** ± 6.1 | 327** ± 6.9 |
|  | E | 0** ± 0.0 | 16** ± 0.3 | 22** ± 0.4 | 31** ± 0.6 | 37** ± 0.7 | 42** ± 0.8 | 55** ± 1.0 | 206** ± 3.7 | 105** ± 1.9 | 133** ± 2.4 |
| Basophils (/µl) | C | 44 ± 0.9 | 39 ± 0.8 | 31 ± 0.6 | 99 ± 2.1 | 55 ± 1.2 | 113* ± 2.4 | 94* ± 1.9 | 72** ± 1.5 | 146** ± 3.1 | 196** ± 4.1 |
|  | E | 18 ± 0.3 | 29 ± 0.5 | 38 ± 0.7 | 39 ± 0.7 | 52 ± 0.9 | 64* ± 1.1 | 75* ± 1.3 | 308** ± 5.5 | 205** ± 3.7 | 76** ± 1.4 |
| Neutrophils (/µl) | C | 4471* ± 59.5 | 4488* ± 59.3 | 7041 ± 93.4 | 6903 ± 91.6 | 5898* ± 78.3 | 6104 ± 81.0 | 7150* ± 94.9 | 8583 ± 113.9 | 8186 ± 108.6 | 6403* ± 84.9 |
|  | E | 5650* ± 50.8 | 6942* ± 62.5 | 7374 ± 66.4 | 7645 ± 68.8 | 8804* ± 79.2 | 7773 ± 69.9 | 5705* ± 51.3 | 9499* ± 85.5 | 9109 ± 81.9 | 7523 ± 67.7 |
| Lymphocytes (/µl) | C | 2554** ± 35.7 | 2852* ± 39.9 | 3003* ± 42.0 | 2465 ± 34.5 | 4686** ± 65.6 | 4635* ± 64.9 | 4945** ± 69.2 | 5150** ± 72.1 | 5701** ± 79.8 | 5880** ± 82.3 |
|  | E | 1470** ± 4.2 | 1690* ± 9.8 | 1909** ± 14.5 | 2802 ± 25.2 | 3110** ± 28.0 | 4280* ± 38.5 | 4850** ± 43.6 | 10953** ± 98.6 | 9355** ± 84.2 | 7054** ± 63.5 |
| Myelocytes (/µl) | C | 162 ± 2.3 | 231** ± 3.2 | 207* ± 2.9 | 99 ± 1.4 | 276* ± 3.7 | 226* ± 3.2 | 94** ± 1.3 | 215** ± 3.0 | 292* ± 4.1 | 261* ± 3.7 |
|  | E | 112 ± 1.0 | 93**± 0.8 | 107*±0.9 | 123 ± 1.1 | 397*± 3.6 | 321*± 2.9 | 225*± 2.0 | 784**±7.1 | 206* ± 1.8 | 104* ± 0.9 |

^1^<1 - sample collected at delivery; 24h - sample collected 24h after delivery; 3d-sample collected 3^rd^ days after delivery; 5d- sample collected 5^th^ days after delivery; 10g - sample collected 10^th^ days after delivery; 20d - sample collected 20^th^ days after delivery; 30d - sample collected 30^th^ days after delivery; 40d - sample collected 40^th^ days after delivery; 50d - sample collected 50^th^ days after delivery; 60d - sample collected 60^th^ days after delivery

^2^ means are reported with their standard errors

Group C – control group; Group E – experimental Biotropina-stimulated group (injection in 35th and 40th days after delivery)

* means in row/line for receptor show significant differences (p<0.05)

** means in row/line for receptor show highly significant statistical differences (p<0.01)
